# Transgressive segregations for agronomic improvement using interspecific crosses between *C*. *arietinum* L. x *C*. *reticulatum* Ladiz. and *C*. *arietinum* L. x *C*. *echinospermum* Davis species

**DOI:** 10.1371/journal.pone.0203082

**Published:** 2018-09-07

**Authors:** Mohar Singh, Savita Rani, Nikhil Malhotra, Gopal Katna, Ashutosh Sarker

**Affiliations:** 1 National Bureau of Plant Genetic Resources Regional Station, Phagli Shimla, India; 2 Department of Plant Biotechnology, CSKHP Agricultural University, Palampur, India; 3 Department of Crop Improvement, CSKHP Agricultural University, Palampur, India; 4 International Centre for Agricultural Research in Dry Areas, South Asia and China Regional Programme, DPS Marg, New Delhi, India; National Institute for Plant Genome Research, INDIA

## Abstract

The wild species of chickpea have tremendous potential for enhancing genetic gains of cultigen and have resistant accessions against major biotic and abiotic stresses. In the present study, two wild annual accessions, one each of *C*. *reticulatum* Ladiz. (ILWC 229) and *C*. *echinospermum* Davis (ILWC 246) were assessed for their agro-morphological features and hybridized with different cultivated varieties (BGD 72, PBG 5, ICKG 96029, Pusa 372 and JG 11) of chickpea. Fertile F_1_ plants were developed as revealed by their normal meiotic chromosomal configuration including high pollen stainability percentage and seed set. The effect of genetic and non-genetic factors on crossability performance with respect to pod and seed set was also evident under two growing conditions of North-Western Indian Himalayas. The segregation analysis using F_2_ phenotypic ratio of some distinct morphological (plant growth habit, stem pigmentation at seedling stage and testa texture) characters indicated their monogenic inheritance pattern. The study would also be useful to chickpea breeders to identify true to type interspecific plants. Further, the F_1_, F_2_ and F_3_ generations of all seven crosses along with parents were evaluated under natural field condition to determine the extent of variability created into the cultivated background of chickpea. There was a wide range of variation in F_3_ population against cold stress, suggesting selection of tolerant recombinant lines at an early stage. We also studied fruitful heterosis (%) as a useful approach, instead of residual heterosis to identify better performing transgressive segregants. The values of most of the interspecific crosses for important traits assessed in F_2_ and F_3_ generations were higher than that of better parent, suggesting isolation of inbred vigour for pod numbers and earliness. The results indicated that wild *Cicer* annual accessions of *C*. *reticulatum* and *C*. *echinospermum* species can be exploited after proper screening for traits of interest for diversification of cultivated gene pool and subsequent use in chickpea improvement.

## Introduction

Chickpea (*Cicer arietinum* L.), is a true diploid (2n = 2x = 16) annual grain legume having genome size of ~738 Mbp [1]. It ameliorates soil fertility through biological nitrogen fixation and also plays a significant role in human diet as a useful source of protein, vitamins and minerals for ever increasing populations in the developing world [2]. As far as chickpea acreage is concerned, it is the second largest pulse crop after dry bean with 14 million hectare area under cultivation [3]. However, the world average yield is 982 kg/ha, which is far below than the actual potential yield of crop [3] because it is generally sown in marginal land with less inputs under rainfed agricultural production system [4]. The traditional breeding methods have not developed much high yielding varieties with large-scale impact on production and productivity of chickpea [5, 6]. Till date, more than 350 improved and 25 mutant varieties have been released by several crop improvement institutions worldwide [7, 8] and the crop has also been classified into desi and kabuli types characterized by its seed size, shape and color [9]. Further, the narrow genetic base of chickpea varieties does not provide much contrasting features for developing improved cultivars [10]. During the process of crop domestication, certain useful productivity genes and alleles were lost, which resulted in narrow genetic base of domesticated species [1, 2, 11–18]. Therefore, chickpea breeders are looking at crop wild relatives (CWRs) as an alternative source of variation for tailoring new germplasm [2, 19]. The genus *Cicer* comprises of 49 taxa [20, 21] and only two wild *Cicer* species, *C*. *reticulatum* Ladiz. and *C*. *echinospermum* Davis are crossable with cultivated gene pool [7]. The wild *Cicer* species consists of useful characters for distinct morphological, agronomical, protein content, and resistance against major biotic and abiotic stresses [22–25]. The present study was, therefore, undertaken to characterize, identify and introgress wild *Cicer* species and appearance of transgression for important quantitative traits, thus exhibiting the favorable effects to determine the extent of variability created into the background of different cultivated chickpea varieties.

## Materials and methods

### Morphological characterization and meiotic study

A total of 88 global wild annual *Cicer* accessions comprising 20 of *C*. *reticulatum* Ladizinsky, 10 of *C*. *echinospermum* P.H. Davis, 25 each of *C*. *judaicum* Boiss. and *C*. *pinnatifidum* Jarb. & Spach, 6 of *C*. *bijugum* Rech. f., and 2 of *C*. *yamashitae* Kitam. were characterized for distinct morphological characters *viz*. plant pigmentation, plant hairiness, number of leaflets leaf^-1^, seed shape, testa texture and seed color. These were also evaluated for important agro-morphological traits (days to flowering, days to maturity, plant height (cm), number of branches plant^-1^, number of pods plant^-1^, number of seeds pod^-1^, 100-seed weight (g), seed yield plant^-1^ (g) and biological yield plant^-1^ (g) in two cropping seasons at two different locations. The wild species were introduced from Biodiversity and Integrated Gene Management (BIGM) Unit at ICARDA, then in Aleppo, Syria during 2010–2011. Further, the characterization and evaluation study has helped us in identifying certain useful accessions including ILWC 229 of *C*. *reticulatum* (resistant to ascochyta blight and desirable pod numbers) and ILWC of *C*. *echinospermum* (resistant to botrytis grey mold and desirable pod numbers) found useful for their introgression into the background of cultivated varieties. Meiotic analysis was carried out by taking appropriate flower buds from the growing plants in pots under green house condition. The flower buds were taken from 25 randomly selected plants of each accession and fixed in Carnoy’s fixative solution (6:3:1 ethanol/chloroform/acetic acid) for 24 h [26]. The collected flower buds were washed properly and preserved in 70% ethanol at 4°C until its use for cytological study. Further, smears of appropriate buds were made in acetocarmine solution [27]. About 25–30 fresh slides of each accession were prepared from different flower buds to draw the valid conclusions. To count chromosome number, about 40 pollen mother cells (PMCs) were observed in different stages at diakinesis/ metaphase-I/anaphase-I and II. Pollen stainability was also assessed by mounting mature pollen grains (PGs) in glycero–acetocarmine (1:1) mixture. About 350–400 pollen grains were analyzed in each case for assessing pollen viability including pollen size. Filled pollen with stained nuclei was recorded as fertile grains, while shrivelled and unstained grains were taken as infertile. Photomicrographs of pollen mother cells and pollen grains were also made from freshly prepared slides using Nikon 80i eclipse Digital Imaging Microscope [26].

### Interspecific hybridization of chickpea

The interspecific hybridization experiments were undertaken at the ICAR-National Bureau of Plant Genetic Resources (NBPGR) Pusa, New Delhi (28^◦^ 35′ N′, 70^◦^ 18′ E, 226m amsl) and the Mountain Agricultural Research and Extension Centre (MAREC) of CSKHPKV, Sangla (31^◦^ 55′ and 32^◦^ 20′ N and 77^◦^ 00′ and 79^◦^ 50′ E, 2,758m amsl) Himachal Pradesh, India. Wild *Cicer* accessions, ILWC 229 (*C*. *reticulatum* Ladiz.) and ILWC 246 (*C*. *echinospermum* Davis) were intercrossed with five released varieties of chickpea *viz*. BGD 72, PBG 5, ICKG 96029, Pusa 372 and JG 11. Total seven interspecific cross-combinations of BGD 72 x ILWC 229, PBG 5 x ILWC 229, BGD 72 x ILWC 246, PBG 5 x ILWC 246, ICKG 96029 x ILWC 246, Pusa 372 x ILWC 246 and JG 11 x ILWC 246 were successfully obtained. To accomplish wide hybridization studies, the emasculation was carried out between 3 to 5 P.M. at both the centers. Pollination was done on next day morning between 8:30 to 10:00 A.M. with mature pollen grains of the male parents as per standard procedure [28, 29]. A solution of growth hormones (GA3-120 ppm + NAA-30 ppm + Kinetin-15 ppm) was also applied to the base of peduncle and pollinated flower buds after pollination to prevent premature pod abscission [29, 30]. The resulted F_1_ seeds of all plants were grown at NBPGR, New Delhi, to obtain F_2_ seeds of each cross-combination during winter season of 2013–2014, and pollen fertility (% stainable pollen) of F_1_ plants was also determined by staining mature pollen grains with 2% acetocarmine solution. However, F_2_ seeds of all crosses were advanced to produce F_3_ seeds and evaluated for important agro-morphological characters in the summer season of 2014 at Sangla.

### Field evaluation of parents and their advanced progenies

The genetic materials comprising of F_1_, F_2_ and F_3_ progenies alongwith parental lines were sown in the Experimental Farm of NBPGR, Regional Station Shimla (31^◦^ 05′ 53′ N and 77^◦^ 09′ 35′ E 1924 m amsl) during winter season of 2015–16. The seeds were planted in 3 m long rows spaced at 10 cm (plant to plant) and 40 cm (row to row) apart. One pre-sowing irrigation was applied to ensure satisfactory seed germination. Recommended cultural practices were followed for raising the genetic materials. During the whole cropping season, one rain and two snow falls was experienced and necessity of manual irrigation was not felt. Five plants of each parental line and F_1_ plants, and all available plants of F_2_ and F_3_ populations were selected for recording data on days to flowering, days to maturity, plant height (cm), number of branches plant^-1^, number of pods plant^-1^, 100-seed weight (g), seed yield plant^-1^ (g), and biological yield plant^-1^ (g). Fruitful heterosis was also calculated following Koseoglu et al. [31] in both F_2_ and F_3_ generations as H_F_ (%) = [(F_2_, F_3_-BP)/BP] × 100, where, F_2_ and F_3_ are generations of interspecific populations and BP is the mean of better parent of a cross.

### Screening of F_3_ progeny against cold tolerance

All F_3_ interspecific plant populations of seven crosses were screened against cold tolerance under natural field condition at Shimla centre. The experiment was conducted in Augmented Block Design [32] in which the best cold tolerant accession, IC31649 was used as standard check repeated after every 25th rows. For visual screening of interspecific plant populations against cold tolerance, a 1–9 rating scale was used [33] where 1- No visible expression of damage; 2- Highly tolerant, up to 10% leaflets shows drying no killing; 3- Tolerant, 11–20% leaflets shows withering, but no killing; 4- Moderately tolerant, 21–40% leaflets shows withering and drying; 5- Intermediate, 41–60% leaflets show withering and drying leaflets, along with 5% plant killing; 6- Moderately susceptible, 61–80% leaflets shows withering and drying symptoms and 6–25% plant killing; 7- Susceptible, 81–99% leaflets shows withering and drying along with 26–50% plant killing; 8- Highly susceptible, 100% leaflets shows withering and drying symptoms, and 51–99% plant killing; 9–100% plants were killed from cold.

Further, observations were recorded three times during the cropping season and all plants were also covered in fresh snow for three days, during winter season of 2016–2017. The lowest average mean temperature (2.8°C) was recorded during flowering stage. The average weather conditions (from October 2016 to April 2017) of cropping period, total rainfall, maximum, minimum temperatures and relative humidity (%) is given in Table 1.

**Table 1:** Total rainfall, maximum, minimum temperatures and relative humidity during cropping period for screening of F_3_ progeny against cold tolerance.

Months	Rainfall (mm)	Temperature (^0^C)		Relative humidity (%)	
		Max	Min	Max	Min
October	2.30	22.90	12.90	60.39	50.32
November	0.00	21.00	10.56	45.70	34.20
December	5.50	19.00	8.38	48.97	36.97
January	148.10	12.50	2.80	69.52	58.00
February	19.60	17.38	6.47	55.32	42.86
March	36.10	25.05	7.77	59.23	48.32
April	85.20	24.78	13.18	55.00	43.10

### Statistical analysis

The genetic materials comprised parental lines and advanced progenies, which were studied for segregation analysis of important distinct morphological characters. Each F_2_ plant was observed for contrasting traits and the chi-square test for goodness of fit was also estimated. The X^2^ value is calculated from observed (O) and expected (E) results from total populations using following formula:

$$X^{2}={\sum \frac{\left(O-E\right)}{E}}^{2}$$

Where, ∑ refers to sum of values of X^2^ over the classes of an experiment. Yate’s factor was also used, where the population size was small. Further, the means were adjusted using online software package for augmented block design (ABD) developed by Rathore et al. [32]. The quantitative characters were further analyzed for various statistical parameters viz. range, mean, coefficient of variation, and fruitful heterosis (%) using the statistical software SYSTAT-12. For screening chickpea F_3_ plant populations against cold tolerance, data were taken using 1–9 rating scale and further analysis following MS Office Excel and SAS software (SAS 2011).

## Results

### Morphological characterization and meiotic study

**Fig 1:**
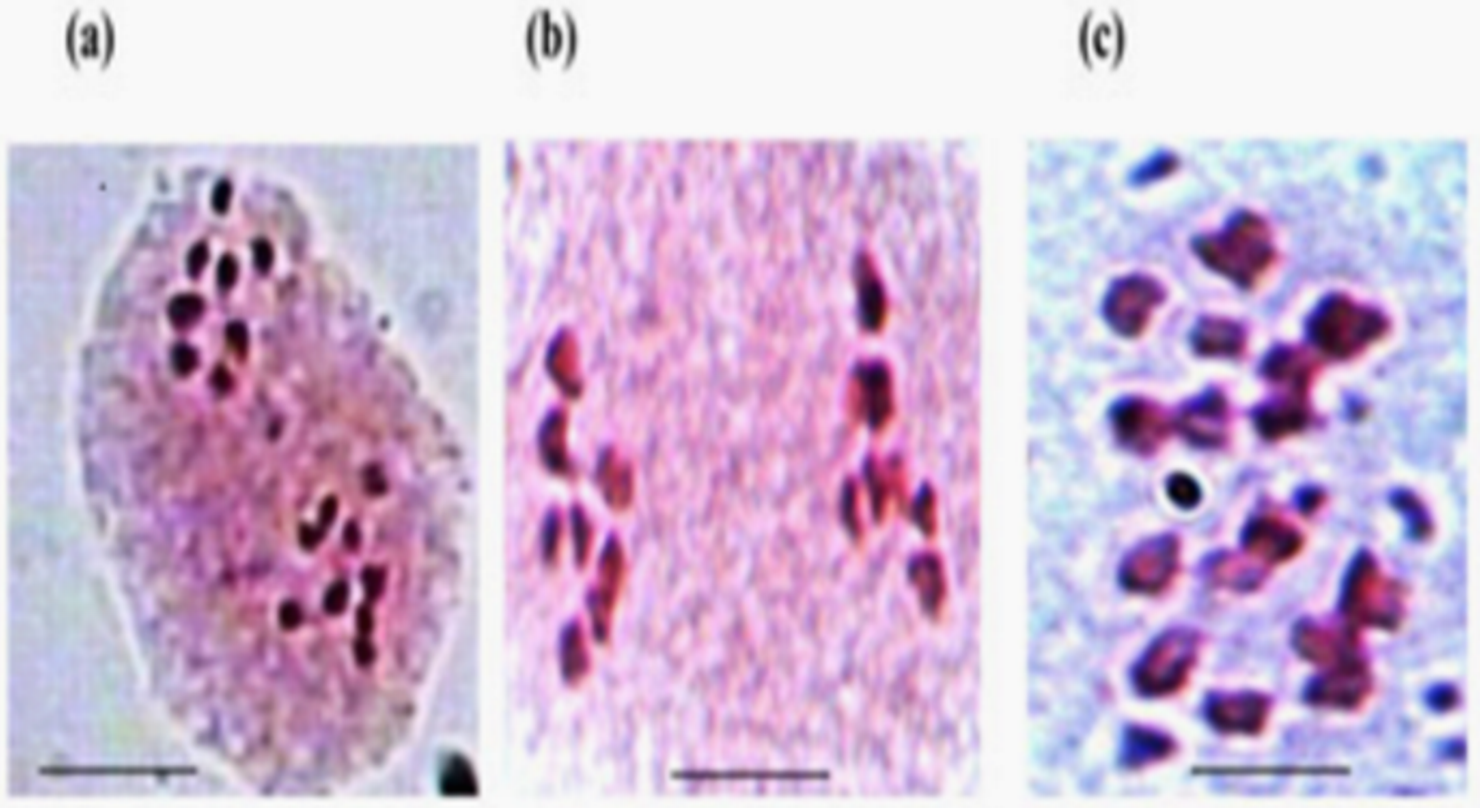
**Pollen mother cells (PMCs) of wild annual *Cicer* species indicates normal breeding behavior where (a) accession ILWC 229 (*C*. *reticulatum*) showing usual separation of chromosomes at anaphase I, (b) accession ILWC 246 (*C*. *echinospermum*) indicates stable late anaphase, (c) accession ILWC 246 (*C*. *echinospermum*) chromosomes arranged in metaphase**. Scale = 10 μm.

**Fig 2:**
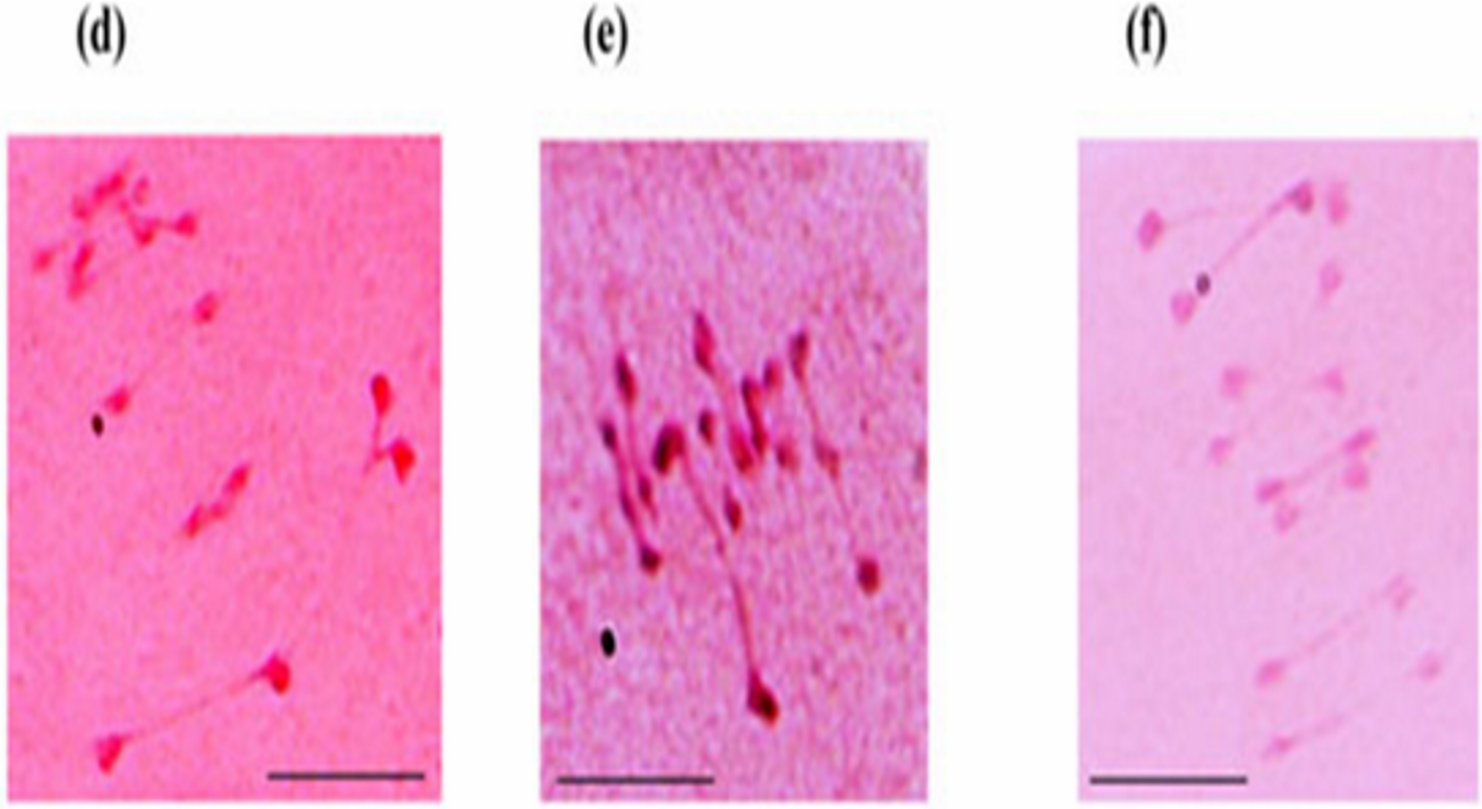
**Meiotic analysis of F_1s_ of (d) *C*. *arietinum* x *C*. *reticulatum* showing normal separation of chromosomes at anaphase II and (e and f) of *C*. *arietinum* x *C*. *echinospermum* exhibiting normal division of chromosomes at metaphase stage**. Scale = 10 μm.

Both the wild annual accessions of *C*. *reticulatum* Ladiz. (ILWC 229) and *C*. *echinospermum* Davis (ILWC 246) were studied for important morphological characters *viz*. plant pigmentation, plant hairiness, number of leaflets leaf^-1^, seed shape, testa texture, flower colour and seed colour. The plant pigmentation showed low anthocyanin in ILWC 229 and no anthocyanin in ILWC 246. Light pubescence on leaves was observed in both the species. The number of leaflets leaf^-1^ was 9–11 in ILWC 229 and 11–13 in ILWC 246. Both the species expressed angular shape of seeds. The texture of testa was rough in ILWC 229 and it was tuberculated in ILWC 246. An accession ILWC 229 produced brown seed, while ILWC 246 black in color. Both the wild species showed normal chromosome pairing producing 8 bivalents (2n = 2x = 16) along with high percentage of pollen stainability. However, its pollen viability percentage ranged from 81.76 (pollen size 22.23 × 17.23 μm) in ILWC 246 to 100 (pollen size 24.23 × 18.56 μm) in ILWC 229 (Fig 1). The F_1_’s of all interspecific cross-combinations showed normal meiosis along with high pollen viability percentage (Fig 2).

### Seed set in F_1_ hybrids

The seed set in F_1_ hybrids under two agro-ecological conditions was recorded with respect to number of cross-pollinations attempted, pod set, seed set, number of seeds pod^-1^ and pollen viability (Table 2). The observations recorded in first set of wide hybridization experiment resulted the production of 54 healthy F_1_ seeds, of 907 cross-pollinations attempted. The maximum seed set (8.03%) was recorded for JG 11 x ILWC 246. The seed set percentage ranged from 4.48 (ICKG 96029 x ILWC 246) to 8.03 (JG 11 x ILWC 246) with an average seed set of 6.44%. Number of seeds pod^-1^ was observed more than one in all F_1_ crosses and pollen viability percentage ranged from 88.12 (ICKG 96029 x ILWC 246) to 93.10 (BGD 72 x ILWC 229). An average maximum/minimum temperature was recorded as 31.4/19.2°C. Identical observations were also reported in the second set of experiment, which resulted in production of 66 F_1_ seeds of 813 cross-pollinations. The highest seed set (10.67%) was observed for PBG 5 x ILWC 229. Pollen stainability percentage ranged from 87.10 (BGD 72 x ILWC 246) to 92.12 (BGD 72 x ILWC 229). The maximum/minimum summer season Himalayan nursery temperature was observed as 26.3/12.5°C. It is summarized that influence of genetic (species/accession groups) and non-genetic factors (day length, growing conditions and temperature) on seed set played a vital role under varied growing conditions.

**Table 2:** Number of cross-pollinations attempted, pod set, seed set, seeds pod^-1^ and pollen viability (%) during winter 2012–13 at New Delhi and summer Himalayan nursery 2013 at Sangla.

		No. of cross-pollinations attempted		Pod set		Seed set		Number of seeds crossed pod^-1^		Pollen viability (%) of F_1_ plants	
S.N.	Cross-combination	Delhi	Sangla	Delhi	Sangla	Delhi	Sangla	Delhi	Sangla	Delhi	Sangla
1	BGD 72 x ILWC 229	150	147	10 (6.66) μ	12 (8.16)	10 (6.66)	12 (8.16)	1.00	1.00	93.10	92.12
2	PBG 5 X ILWC 229	114	103	09 (7.89)	10 (9.70)	08 (7.01)	11 (10.67)	1.12	1.10	91.10	90.11
3	BGD 72 X ILWC 246	112	105	06 (5.35)	10 (9.52)	06 (5.35)	07 (6.66)	1.00	0.70	90.11	87.10
4	PBG 5 X ILWC 246	111	110	06 (5.40)	10 (9.09)	07 (6.30)	09 (8.18)	1.16	1.11	89.10	89.12
5	ICKG 96029 X ILWC 246	156	113	08 (5.12)	09 (7.96)	07 (4.48)	09 (7.96)	1.14	1.00	88.12	90.00
6	PUSA 372 X ILWC 246	152	119	09 (5.92)	11 (9.24)	07 (4.60)	09 (7.56)	1.28	1.22	91.11	88.88
7	JG 11 X ILWC 246	112	116	09 (8.03)	11 (9.82)	09 (8.03)	09 (7.75)	1.00	1.22	90.12	90.10
Average performance		129.57	116.14	8.14 (6.42)	10.42 (9.15)	7.71 (6.44)	9.42 (8.60)	1.09	1.02	90.03	89.63

*μ Percentage of cross-pollinations attempted in parenthesis; Average max/min winter temperature during whole crossing period 31.4/ 19.2°C at Delhi; Average max/min summer temperature during whole crossing period 26.3/12.5°C at Sangla

## Inheritance of distinct morphological traits

### Plant growth habit

Two interspecific crosses were made between PBG 5 (erect growth habit) and ILWC 229 and ILWC 246 having spreading growth habit. The F_1_ plants showed erect growth indicating that erect plant growth is dominant over spreading habit. In F_2_ generation, 373 plants showed erect plant type and 144 plants showed spreading habit for cross PBG 5 x ILWC 229, while 181 plants showed erect plant type and 78 showed spreading growth habit for cross PBG 5 x ILWC 246 (Table 3).

**Table 3:** Inheritance pattern of distinct morphological traits in cultivated x wild chickpea crosses.

Trait	Cross	Generation	Observed progeny	Expected ratio	Chi-square	P value
Plant growth habit	PBG 5 × ILWC 229	F_1_	18 (erect)	-	-	-
		F_2_	373 (erect) : 144 (spreading)	3:1	2.24	0.2–0.3
	PBG 5 × ILWC 246	F_1_	20 (erect)	-	-	-
		F_2_	181(erect) : 78 (spreading)	3:1	2.61	0.3–0.5
Stem pigmentation at seedling stage	Pusa 372 x ILWC 229	F_1_	18 (pigmented)	-	-	-
		F_2_	172 (pigmented) : 114 (non-pigmented)	9:7	1.76	0.3–0.5
	PBG 5 x ILWC 229	F_1_	16 (pigmented)	-	-	-
		F_2_	276 (pigmented) : 241 (non-pigmented)	9:7	1.72	0.2–0.5
Testa Texture	Pusa 256 x ILWC 229	F_1_	20 (rough)	-	-	-
		F_2_	94 (rough) : 33 (smooth)	3:1	0.07	0.3–0.5
	BGD 72 x ILWC 229	F_1_	18 (rough)	-	-	-
		F_2_	82 (rough) : 34 (smooth)	3:1	1.15	0.2–0.3

### Stem pigmentation at seedling stage

The stem pigmentation at seedling stage was studied in two interspecific crosses of Pusa 372 x ILWC 229 and PBG 5 x ILWC 229. The F_1_ plants expressed pigmented stems at seedling stage indicating that the trait is dominant over normal or non-pigmented, and in F_2_ generation of both crosses, the trait segregated into 9 pigmented: 7 non-pigmented ratio (Table 3).

### Testa texture

The trait testa texture was studied in two interspecific crosses of Pusa 256 x ILWC 229 and BGD 72 x ILWC 229. The F_1_ hybrids of each cross exhibited rough texture, indicating that it is dominant over smooth (Table 3) and F_2_ generation of both crosses segregated into rough and smooth seeds fitting well into the ratio of 3 rough : 1 smooth texture pattern.

### Field evaluation of parental lines and their advanced progenies

**Fig 3:**
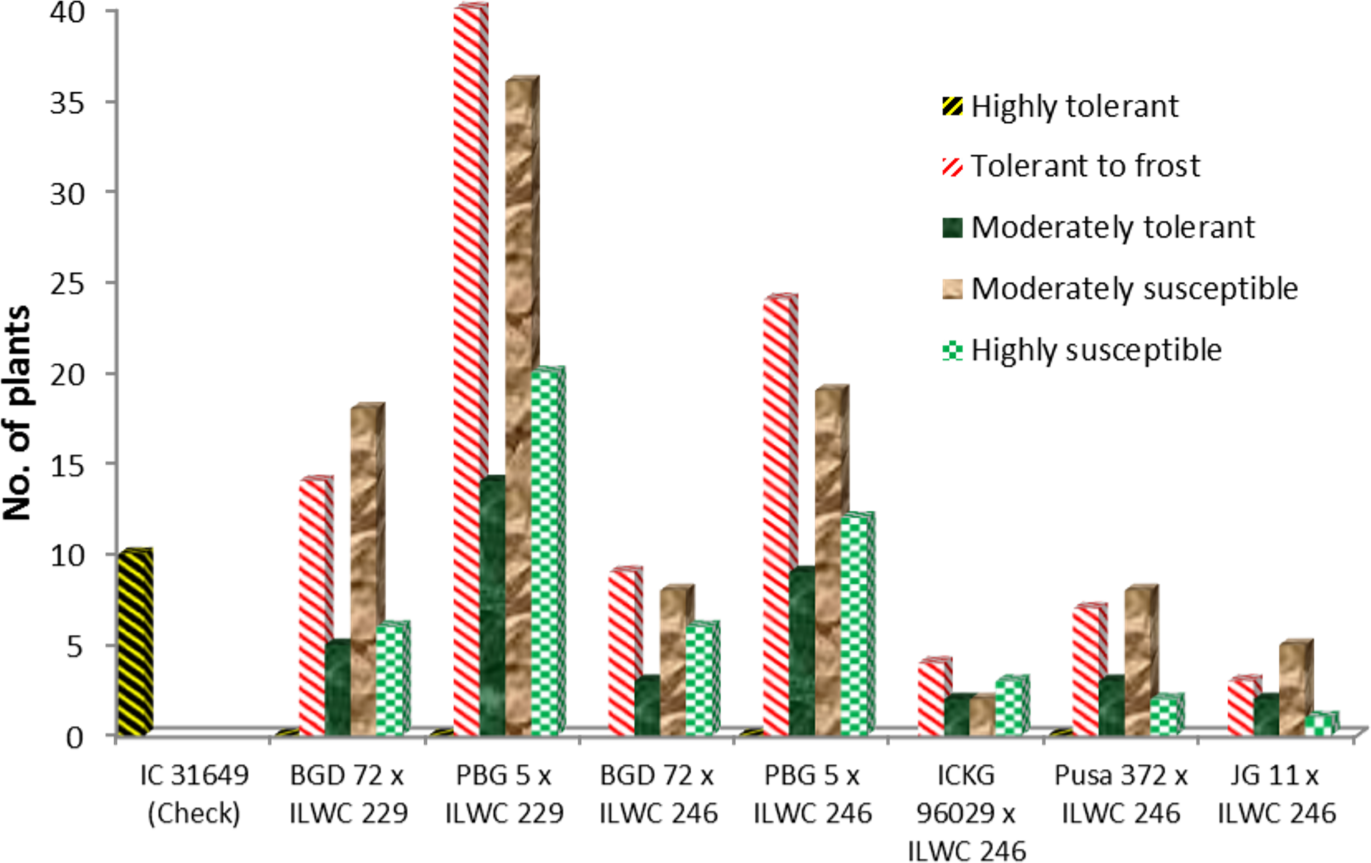
Distribution of F_3_ interspecific populations of different crosses into various reactions against cold tolerance.

The parental lines, their F_1s_, F_2s_ and F_3s_ were evaluated under natural field conditions to assess the variability created through transgressive segregants of various interspecific crosses (Table 4). The F_1s,_ involving early flowering and maturing cultivated parents (BGD 72, PBG 5, ICKG 96029, Pusa 372 and JG 11) and late flowering wild accessions (ILWC 229 and ILWC 246) showed interesting comparability. For the trait plant height, all F_1_ plants were tall for majority of crosses_,_ and in F_2_ and F_3_ generations, it revealed variability through segregations towards dwarf to taller plants. However, number of pods plant^-1^ also manifested desirable performance for majority of F_1_ crosses, and in F_2_ and F_3_ generations, a wide range of variation occurred in all wide cross-combinations such as plant height, number of branches^-1^, number of pods^-1^ and seed yield plant^-1^. The seed yield plant^-1^, exhibited desirability in F_1_ hybrids, while in F_2_, and F_3_ derivatives, a substantial variation did appear in majority of interspecific cross-combinations of BGD 72 x ILWC 229, PBG 5 x ILWC 229, BGD 72 x ILWC 246, PBG 5 x ILWC 246, ICKG 96029 x ILWC 246 and Pusa 372 x ILWC 246. Likewise, for biological yield plant^-1^, F_1_ hybrids of almost all crosses manifested high biomass score as compared to their parents, and in F_2_ and F_3_ generations, variability appeared for this trait from low to high biomass yield. Furthermore, the F_3_ plant populations of all interspecific crosses were screened against cold tolerance and results showed that in each cross-combination, some interspecific recombinant lines exhibited complete tolerance against cold stress. The maximum number of plants were observed tolerant against cold stress in cross- combination of PBG 5 x ILWC 229 followed by PBG 5 x ILWC 246 and BGD 72 x ILWC 229. None of the plants were recorded highly tolerant against cold stress under natural field condition. An average mean temperature for the whole cropping season is presented in Table 1 and the distribution of plant populations of each interspecific cross in different resection against cold is given in Fig 3.

**Table 4:** Range, mean, standard error and coefficient of variation for agro-morphological traits in different generations of crosses involving cultivated x wild chickpea.

Character	P_1_			P_2_			F_1_			F_2_			F_3_		
	Range	Mean±SE	CV (%)	Range	Mean±SE	CV (%)	Range	Mean±SE	CV (%)	Range	Mean±SE	CV (%)	Range	Mean±SE	CV (%)
**Days to flowering**													** **	** **	** **
BGD 72 x ILWC 229	62.0–72.0	67.3±2.9	7.4	75–80	77.5±2.5	4.5	78.1–89	84.0±0.2	13.7	68–91	76.1±2.7	10.3	83.0–92.0	87.1±0.41	2.13
PBG 5 x ILWC 229	67.0–71.0	69.0±1.1	2.8	75–80	77.5±2.5	4.5	72.0–86.0	80.3±2.4	8.6	55–117	75.0±1.8	17.9	81.0–89.0	84.2±0.25	2.15
BGD 72 x ILWC 246	62.0–72.0	67.3±2.9	7.4	65–70	67.3±2.1	3.7	80.0–89.0	86.8±3.4	7.9	67–98	79.8±4.5	14.9	83.0–88.0	85.7±0.41	1.73
PBG 5 x ILWC 246	67.0–71.0	69.0±1.1	2.8	65–70	67.3±2.1	3.7	90.0–96.0	93.7±3.6	6.6	60–115	83.4±3.1	20.3	89–99.0	96.3±0.40	2.43
ICKG 96029 x ILWC 246	57.0–66.0	60.6±2.7	7.7	65–70	67.3±2.1	3.7	95.0–102	99.0±0.5	0.9	57–108	72.5±6.8	24.8	92.0–104	102.8±1.55	1.60
PUSA 372 x ILWC 246	64.0–67.0	65.3±0.8	2.3	65–70	67.3±2.1	3.7	89.0–96.0	93.0±0.5	0.9	75.0–96.0	84.5±5.0	15.6	85.8–93.6	89.6±0.70	2.52
JG 11 x ILWC 246	56.0–67.0	60.6±3.2	9.3	65–70	67.3±2.1	3.7	87.0–99.9	93.0±0.5	0.7	65.0–93.0	80.3±7.4	22.5	82.0–97.2	94.5±0.50	1.39
**Days to maturity**													** **	** **	** **
BGD 72 x ILWC 229	114.0–122.0	117.6±2.3	2.9	125–135	130.0±2.0	8.2	110–152	146.6±0.6	0.6	115–132	124.1±6.2	9.1	114–125	119.1 ± 0.57	1.84
PBG 5 x ILWC 229	125.0–130.0	122.6±1.4	1.4	125–135	130.0±2.0	8.2	116–126	112.0±1.5	4.1	116–140	132.9±3.2	15.2	123–135	129.3 ± 0.29	1.55
BGD 72 x ILWC 246	114.0–122.0	117.6±2.3	2.9	115–131	127.6±1.4	1.4	140–154	151.2±0.7	1.0	135–142	139.1±2.1	3.2	128–150	143.8 ± 0.60	1.58
PBG 5 x ILWC 246	125.0–130.0	117.6±1.4	1.4	115–131	127.6±1.4	1.4	110–126	121.1±0.7	1.3	119–128	122.0±3.1	12.1	125–132	119.1 ± 0.26	1.01
ICKG 96029 x ILWC 246	143.0–148.0	145.3±1.4	1.7	115–131	127.6±1.4	1.4	119–125	120.0±0.5	0.5	114–147	134.7±5.4	10.6	113–141	136.9 ± 0.83	1.25
PUSA 372 x ILWC 246	145.0–149.0	146.6±1.2	1.4	115–131	127.6±1.4	1.4	120–127	123.0±1.7	1.7	121–138	133.2±9.5	16.4	115–139	123.2 ± 0.91	2.1
JG 11 x ILWC 246	131.0–175.0	146.0±14.5	17.1	115–131	127.6±1.4	1.4	120–127	121.3±0.8	0.8	112–131	117.0±5.3	9.4	124–146	137.3± 0.82	1.44
**Plant height (cm)**															
BGD 72 x ILWC 229	40.0–52.0	44.6±3.7	14.3	29.0–51.0	40.0±11.0	38.8	68.0–76.0	71.3±2.4	5.8	30.0–63.0	39.6±3.6	25.8	34.8–65.2	52.9 ±1.28	15.5
PBG 5 x ILWC 229	17.0–22.0	19.6±1.4	12.7	29.0–51.0	40.0±11.0	38.8	53.0–84.0	64.7±1.7	11.4	8.0–87.0	56.8±2.2	28.6	34.2–72.4	52.6 ±0.92	18.4
BGD 72 x ILWC 246	40.0–52.0	44.6±3.7	14.3	41.0–50.0	44.6±2.7	10.5	57.0–65.0	60.8±1.3	4.8	32.0–70.0	51.5±5.6	28.8	38.4–69.4	53.4±1.63	15.1
PBG 5 x ILWC 246	17.0–22.0	19.6±1.4	12.7	41.0–50.0	44.6±2.7	10.5	42.0–84.0	68.5±5.6	23.2	36.0–81.0	55.8±3.1	30.7	38.3–78.4	58.5 ±1.26	17.2
ICKG 96029 x ILWC 246	57.0–63.0	60.0±1.7	5	41.0–50.0	44.6±2.7	10.5	48.0–52.0	50.0±1.1	4.0	28.0–83.0	54.0±8.4	41.5	37.8–70.8	56.1±3.30	19.5
PUSA 372 x ILWC 246	28.0–41.0	34.6±3.7	18.7	41.0–50.0	44.6±2.7	10.5	51.0–54.0	52.3±0.8	2.9	43.0–82.0	55.4±5.2	25.2	32.4–59.5	40.1±1.31	14.7
JG 11 x ILWC 246	65.0–71.0	68.3±1.7	4.4	41.0–50.0	44.6±2.7	10.5	56.0–63.0	59.0±2.0	6.1	45.0–67.0	53.8±3.8	17.2	34.4–53.8	42.3±1.74	14.3
**No. of branches plant**^**-1**^															
BGD 72 x ILWC 229	4.0–7.0	5.6±0.8	26.8	9.0–12.0	10.5±1.5	20.1	10.0–15.0	12.6±1.4	19.8	2.0–12.0	7.0±1.2	48.8	7.0–23.0	15.4±0.57	23.6
PBG 5 x ILWC 229	7.0–11.0	9.0±1.1	22.2	9.0–12.0	10.5±1.5	20.1	6.0–19.0	12.0±0.8	29.4	3.0–37.0	13.3±0.9	50.2	7.0–25.0	15.8±0.35	22.9
BGD 72 x ILWC 246	4.0–7.0	5.6±0.8	26.8	8.0–14.0	11.0±1.7	27.2	13.0–30.0	19.6±2.8	32.9	8.0–16.0	11.4±1.3	31.8	7.0–19.0	12.3±0.51	21.2
PBG 5 x ILWC 246	7.0–11.0	9.0±1.1	22.2	8.0–14.0	11.0±1.7	27.2	6.0–17.0	10.8±1.2	32.3	3.0–23.0	9.4±0.8	47.2	7.0–24.0	14.2±0.48	26.9
ICKG 96029 x ILWC 246	3.0–6.0	4.3±0.8	35.1	8.0–14.0	11.0±1.7	27.2	16.0–21.0	19.0±1.5	13.8	4.0–12.0	8.0±1.0	34.5	7.0–18.0	10.9±1.05	31.9
PUSA 372 x ILWC 246	8.0–9.0	8.6±0.3	6.5	8.0–14.0	11.0±1.7	27.2	23.0–27.0	25.3±1.2	8.2	9.0–30.0	15.5±2.6	44.7	9.0–19.0	14.2±0.66	20.7
JG 11 x ILWC 246	10.0–15.0	12.3±1.4	20.3	8.0–14.0	11.0±1.7	27.2	19.0–24.0	22.0±1.5	12	4.0–23.0	12.6±2.8	55.5	7.0–21.0	15.1±1.13	25.7
**No. of pods plant**^**-1**^															
BGD 72 x ILWC 229	13.0–16.0	14.3±0.8	10.6	7.0–9.0	8.0±1.0	17.6	25.0–51.0	33.6±8.6	44.5	10.0–35.0	21.2±2.9	39.7	50–104	71.3± 2.27	20.38
PBG 5 x ILWC 229	22.0–24.0	23.0±0.5	4.3	7.0–9.0	8.0±1.0	17.6	4.0–58.0	24.9±3.0	54	5.0–165.0	25.8±4.1	113.2	41–112	78.0± 1.45	19.53
BGD 72 x ILWC 246	13.0–16.0	14.3±0.8	10.6	3.0–7.0	4.6±1.2	44.6	27.5–75.0	42.4±8.7	45.8	2.0–27.0	13.2±3.9	78.9	43–80	59.1± 1.83	15.81
PBG 5 x ILWC 246	22.0–24.0	23.0±0.5	4.3	3.0–7.0	4.6±1.2	44.6	1.5–40.0	25.7±5.4	60.1	2.0–74.0	17.0±3.3	106.5	50–135	71.2± 2.16	24.26
ICKG 96029 x ILWC 246	16.0–19.0	17.6±0.8	8.6	3.0–7.0	4.6±1.2	44.6	32.0–35.0	34.0±1.0	5.0	4.0–86.0	26.8±10.5	103.3	24–79	51.2± 4.25	27.49
PUSA 372 x ILWC 246	16.0–25.0	19.6±2.7	24	3.0–7.0	4.6±1.2	44.6	37.0–39.0	38.0±0.5	2.6	2.0–105.0	37.5±13.8	97.5	39–91	64.9±3.04	20.94
JG 11 x ILWC 246	47.0–94.0	69.6±13.6	33.7	3.0–7.0	4.6±1.2	44.6	30.0–32.0	31.0±0.5	3.2	5.0–25.0	11.8±2.9	61.2	46–79	58.2±2.77	16.5
**100-seed weight (g)**															
BGD 72 x ILWC 229	26.0–28.0	27.0±0.5	3.7	11.0–12.0	11.5±0.4	6.0	18.4–24.2	20.3±1.9	16.4	16.0–45.0	23.1±3.4	41.6	11.1–28.7	17.8±0.92	32.87
PBG 5 x ILWC 229	13.5–17.5	15.5±1.1	12.9	11.0–12.0	11.5±0.4	6.0	2.0–18.6	14.9±1.0	28.7	9.0–24.0	14.4±0.4	20.4	10.6–23.6	17.6±0.25	14.68
BGD 72 x ILWC 246	26.0–28.0	27.0±0.5	3.7	7.1–7.8	7.4±0.2	0.1	12.8–21.2	17.6±1.9	25.0	15.0–20.8	18.2±0.9	13.2	12.6–22.6	17.8±0.41	11.86
PBG 5 x ILWC 246	13.5–17.5	15.5±1.1	12.9	7.1–7.8	7.4±0.2	0.0	7.6–19.0	12.9±2.1	39.7	12.0–20.5	15.8±0.4	15.9	12.4–27.8	18.4±0.38	16.36
ICKG 96029 x ILWC 246	13.0–28.0	21.6±4.4	35.8	7.1–7.8	7.4±0.2	0.0	16.3–16.4	16.3±0.1	0.3	8.0–18.4	13.4±1.7	34.1	13.6–21.6	18.0±0.87	13.7
PUSA 372 x ILWC 246	12.0–14.8	12.9±0.9	12.4	7.10–7.8	7.46±0.2	0.02	17.6–17.7	17.6±0.1	0.2	13.6–15.0	14.4±0.2	3.6	13.8–19.8	16.5±0.38	10.28
JG 11 x ILWC 246	19.2–21.2	20.0±0.6	5.2	7.1–7.8	7.4±0.2	0.1	18.4–18.5	18.4±0.1	0.2	1.8–20.0	4.2±2.6	45.8	11.0–18.2	14.7±0.65	15.2
**Seed-yield plant**^**-1**^ **(g)**															
BGD 72 x ILWC 229	2.7–3.0	2.8±0.2	5.3	0.8–2.7	1.7±0.9	76.5	4.6–15.7	8.3±3.6	77.1	1.8–6.0	3.6±0.5	41.8	5.6–19.9	12.9 ± 0.56	27.94
PBG 5 x ILWC 229	3.5–4.1	3.8±0.1	7.8	0.8–2.7	1.7±0.9	76.5	0.1–6.4	4.3±0.4	42.5.	0.6–25.4	4.2±0.6	108.9	6.1–27.4	13.9 ± 0.42	31.77
BGD 72 x ILWC 246	2.7–3.0	2.8±0.4	5.3	0.3–1.0	0.5±0.2	66	4.6–11.4	7.1±1.2	38.9	0.2–6.7	2.5±1.0	103.8	6.1–16.5	10.9 ± 0.57	26.68
PBG 5 x ILWC 246	3.5–4.1	3.8±0.1	7.8	0.3–1.0	0.5±0.2	66	0.1–7.7	5.5±2.0	46.7	0.2–17.4	3.8±0.8	117.8	6.2–26.4	13.3 ± 0.53	31.67
ICKG 96029 x ILWC 246	3.0–3.6	3.2±0.1	9.2	0.3–1.0	0.5±0.2	66	4.5–4.6	4.5±0.1	1.0	0.4–11.4	3.7±1.3	97	3.4–16.3	10.0±1.37	38.68
PUSA 372 x ILWC 246	2.6–4.6	3.6±0.5	27.5	0.3–1.0	0.5±0.2	66	5.2–5.3	5.2±0.1	0.8	0.2–14.9	5.9±2.1	93.7	7.9–14.8	11.3 ± 0.43	16.89
JG 11 x ILWC 246	13.2–21.5	16.7±2.4	25.4	0.3–1.0	0.5±0.2	66	5.0–5.1	5.0±0.1	0.3	1.0–3.8	1.9±0.4	58.8	6.1–13.4	8.7 ±0.56	22.16
**Biological yield plant**^**-1**^ **(g)**															
BGD 72 x ILWC 229	3.9–4.9	4.3±0.3	12	2.2–4.10	3.1±0.95	42.5	13.4–34.2	20.3±6.9	59	4.2–27.9	12.4±2.5	56.9	16.6–51.8	39.6±1.17	18.9
PBG 5 x ILWC 229	18.8–21.0	20.1±0.6	5.7	2.2–4.10	3.1±0.95	42.5	10.4–22.1	15.2±0.6	19.9	2.4–53.6	16.2±1.3	60.4	24.6–65.6	40.0±0.69	18.1
BGD 72 x ILWC 246	3.9–4.9	4.3±0.3	12	10.1–12.9	11.7±0.8	12.3	18.8–43.3	24.7±4.6	42.2	8.9–22.8	15.5±2.1	37	27.9–51.2	37.8±0.97	13.1
PBG 5 x ILWC 246	18.8–21.0	20.1±0.6	5.7	10.1–12.9	11.7±0.8	12.3	9.8–20.7	14.2±1.6	32	2.7–43.6	15.3±2.0	70.5	25.9–64.6	41.8±1.06	20.3
ICKG 96029 x ILWC 246	3.5–4.2	3.8±0.2	9	10.1–12.9	11.7±0.8	12.3	18.0–18.6	18.3±0.1	1.6	3.6–20.7	10.4±2.6	65.9	23.6–50.9	35.8±2.58	20.4
PUSA 372 x ILWC 246	7.0–12.4	9.5±1.5	28.6	10.1–12.9	11.7±0.8	12.3	34.1–34.9	34.5±0.2	1.1	15.7–44.1	29.2±3.9	35.2	29.8–46.6	38.3±1.06	12.3
JG 11 x ILWC 246	17.3–26.3	21.3±2.6	21.3	10.1–12.9	11.7±0.8	12.3	21.0–22.1	21.4±0.3	2.7	4.5–20.8	13.3±2.7	49.9	31.1–44.6	35.2±1.11	10.9

### Estimates of fruitful heterosis (%)

The nature and magnitude of fruitful heterosis for inbred vigour was studied in F_2_ and F_3_ interspecific derivatives for seed yield plant^-1^ and its important component traits (Table 5). An extent of fruitful heterosis as a vigour was estimated as percent of deviation of enhanced progenies from the better performing parent. In F_2_ generation, the mean performance ranged from -400.70% for plant height (ICKG 96029 x ILWC 246) to 31.02% for number of pods plant^-1^ (BGD 72 x ILWC 229). Likewise, in F_3_ generation, the vigour ranged from -36.82% for plant height (Pusa 372 x LWC 246) to 45.38% for seed yield plant^-1^ (JG 11 x ILWC 246). There were wide range of variation with respect to fruitful heterosis (inbred vigour) for important traits of interest in both F_2_ and F_3_ generations. For majority of interspecific crosses in both the generations, number of pods plant^-1^ and seed yield plant^-1^ revealed positive vigour (Table 5).

**Table 5:** Estimates of fruitful heterosis (%) as inbred vigour for important traits of interest in F_2_ and F_3_ wide cross populations.

Trait/Cross	Generation			
	F_2_		F_3_	
	Range	Mean±SE	Range	Mean±SE
**Pusa 372 x ILWC 246**				
Days to maturity	1.14–6.29	4.26±0.34	5.71–13.71	10.40±0.52
Plant height (cm)	-45.20-(-28.82) μ	-36.80±0.98	-48.96-(-6.30)	-36.82±2.08
No. of pods plant^-1^	-88.33-(-75.00)	-81.25±0.75	-35-(51.67)	8.17±5.06
Seed yield plant^-1^ (g)	-42.75-(-10.14)	-30.33±2.23	-7.25-(2.75)	13.88±3.10
**JG 11 x ILWC 246**				
Days to maturity	5.06–8.43	6.84±0.28	8.99–14.04	10.86±0.46
Plant height (cm)	-31.12-(-13.28)	-20.00±1.80	-28.63-(+5.33)	-12.15±3.62
No. of pods plant^-1^	11.54–30.77	20.67±2.09	-38.67-(+5.33)	-22.33±3.70
Seed yield plant^-1^ (g)	2.44–20.73	10.26±1.67	29.35–85.87	45.38±4.25
**BGD 72 x ILWC 246**				
Days to maturity	-2.47-(+3.70)	0.33±0.39	16.05–23.46	19.66±0.37
Plant height (cm)	-25.56-(+13.69)	-8.58±2.06	-29.93-(+26.64)	-2.52±2.97
No. of pods plant^-1^	-39.47-(+2.63)	-21.58±2.14	3.45–37.93	12.14±1.77
Seed yield plant^-1^ (g)	-31.93-(+2.52)	-19.66±2.16	2.94–61.76	21.30±3.30
**ICKG 96029 x ILWC 246**				
Days to maturity	-6.71-(-0.61)	-4.01±0.93	11.59–16.46	13.97±0.51
Plant height (cm)	-670.73-(-60.98)	-400.70±94.89	-46.15-(+0.85)	-19.98±5.52
No. of pods plant^-1^	7.41–31.48	17.20±3.91	0.01–46.30	11.11±4.50
Seed yield plant^-1^ (g)	0.01–5.49	3.30±0.68	11.10–79.12	27.87±6.74
**PBG 5 x ILWC 246**				
Days to maturity	-11.60-(-2.26)	-7.54±0.48	13.26–17.68	15.57±0.15
Plant height (cm)	-37.21–3.16	-13.51±1.96	-36.38–30.22	-2.74±2.10
No. of pods plant^-1^	0.01–12.86	5.30±0.73	0.01–92.86	15.76±2.07
Seed yield plant^-1^(g)	0.01–16.96	5.93±0.89	0.89–135.71	29.74±3.50
**BGD 72 x ILWC229**				
Days to maturity	1.18–1.18	4.33±1.69	7.2–13.3	10.03±0.32
Plant height (cm)	-54.54-(-5.54)	8.36±2.40	-75.3–81.4	18.46±6.62
No. of pods plant^-1^	1.96–1.96	31.02±5.83	0.01–57.6	15.78±2.24
Seed yield plant^-1^ (g)	2.25–2.25	25.94±4.27	2.00–97.0	34.68±4.02
**PBG 5 x ILWC 229**				
Days to maturity	-2.31–0.82	0.82±0.36	7.82–14.83	11.39±0.16
Plant height (cm)	-32.19–7.37	-7.37±1.63	-34.73–38.17	0.54±1.77
No. of pods plant^-1^	5.00–14.03	14.03±1.29	-1.33–944.00	20.40±8.55
Seed yield plant^-1^ (g)	2.94–17.17	17.17±1.87	-12.93–136.21	30.49±2.91

*μ negative range performance recorded in parentheses

## Discussion

The narrow crop genetic base is forcing plant breeders to search for new adaptive traits of interest. The introduction, characterization, evaluation, identification, and utilization of unadapted gene sources for useful traits are prerequisites conducting successful base broadening programme in annual crop plants [34–36]. The results pertaining to chickpea interspecific hybridization accomplished under two growing seasons help us to conclude those longer days during summer season and optimum temperature has pivotal role in pod and seed setting. Singh et al. [30] have also observed identical role of these factors in determining the onset of ontogenesis in chickpea. The remarkable variation assessed in morphological traits, which can help to distinguish phenotypic groups and their segregation analysis and genetic controls can be established using Mendelian genetic studies. It also permits us in the phenotypic identification of specific alleles for specific gene loci [37,38]. The normal breeding behaviour (meiotic chromosome configuration) of both wild *Cicer* annual accessions and their F_1_ hybrids was confirmed from complete pairing of chromosomes in bivalent forms and the production of fertile off-springs, consequently regular segregation and elite random selection [39,40]. The segregation of some important morphological traits suggested their monogenic inheritance for plant growth habit, stem pigmentation at seedling stage and testa texture. The study would be useful in the identification of true to type F_1_ hybrids through distinct morphological characters.

Further, field evaluation of parents and their advanced progenies using range, mean and coefficient of variation revealed creation of desired variability exhibiting inclusion of useful genes and alleles and possibly major role of complementary gene action [41]. Plant height manifested taller plants in majority of F_1_ hybrids, and in F_2_ and F_3_ generations, variation appears for dwarf to taller plants offering ample scope for selection of elite recombinant lines. Further, desirable plants were recorded in F_1_ generation for high number of pods plant ^-1^ and in F_2_ and F_3_, the trait appear with wide range of variation from low to high pod number, suggesting that *C*. *reticulatum* accession ILWC 229 was better for number of pods plant^-1^, offering possibilities of recovering better plant types with high yield potential in subsequent advanced generations [5, 21, 30]. For seed yield plant^-1^, desirable performance appears for most of crosses in F_1_ generation, and in F_2_ and F_3_ interspecific populations, substantial range of variation was assessed for this trait, suggesting the scope of improvement through single plant selection from F_2_ onwards [5]. Considering linkage drag as a barrier in interspecific hybrid populations, the generation advancement and useful selection in segregating populations is very important to select promising recombinants and therefore, F_2_ population should be adequate in size [5] beside many other useful traits can also be taken well from the segregating plant populations. It further advocated that high yielding lines could be developed from interspecific hybridization following single plant selection [7,42]. The presence of elite transgression for seed yield and other agronomic traits indicating genetic complementarity between recipient and donor parental genotypes [43, 44]. This foster better hope for the recovery of desirable alleles [5, 7, 42]. Further, heterosis breeding has fastened the genetic improvement of crop plants. The consistency in the magnitude of fruitful heterosis in F_2_ and F_3_ generations might be due to accumulation of favorable additive alleles. Such segregants may be tackled as suggested by Redden and Jensen [45] for selecting elite recombinant lines for the development of suitable genotypes. Furthermore, the F_3_ interspecific derivatives also exhibit tolerance against cold stress, such tolerance could be useful selection criteria for developing suitable genotypes or breeding populations for colder areas. Here the prime aim of our study was to characterize, evaluate, and identify useful traits of interest from wild *Cicer* accessions and their introgression for diversification of genetic base of cultivated gene pool. The interspecific hybridization between *C*. *arietinum* L. x *C*. *reticulatum* and *C*. *arietinum* L. x *C*. *echinospermum* crosses showed substantially higher variation for important agro-morphological traits, which offers scope for isolation of potential transgressive segregants for developing high yielding lines or useful donors for further practical breeding purposes [46]. The study would also be useful to the chickpea researchers, while planning their experiments for introgressing useful traits of interest from wild *Cicer* species. The genetic materials are being advanced for further breeding and desirable selection.
